# Role of cervical dendritic cell subsets, co-stimulatory molecules, cytokine secretion profile and beta-estradiol in development of sequalae to Chlamydia trachomatis infection

**DOI:** 10.1186/1477-7827-6-46

**Published:** 2008-10-01

**Authors:** Tanvi Agrawal, Vikas Vats, Paul K Wallace, Sudha Salhan, Aruna Mittal

**Affiliations:** 1 Institute of Pathology (ICMR), Safdarjung Hospital Campus, New Delhi, India; 2 Department of Flow Cytometry, Roswell Park Cancer Institute, Buffalo, USA; 3 Department of Obstetrics and Gynaecology, Safdarjung Hospital, New Delhi, India

## Abstract

**Background:**

Chlamydia trachomatis infection of the female genital tract can lead to serious sequelae resulting in fertility related disorders. Little is known about the mechanism leading to Chlamydia induced pathology and factors responsible for it. As only some of the women develops reproductive disorders while majority of the women clears infection without any severe sequalae, mucosal immune response in women with or without fertility disorders was studied to identify factors which may lead to final clinical outcome of chlamydial infection.

**Methods:**

Myeloid DCs (mDCs) and plasmacytoid DCs (pDCs) populations in cervical mucosa and peripheral blood were analyzed in controls and Chlamydia positive women with or without fertility disorders with multicoloured flow cytometric analysis. Cervical cytokines (IL-6, IL-8, IL-10, IL-12, TNF-alpha and IFN-gamma), C-reactive protein levels and sex hormone levels in serum were quantified by ELISA.

**Results:**

In cervix of Chlamydia positive women with fertility disorders, significantly high (P < 0.05) numbers of pDCs were present with increased CD80 expression. pDCs correlated significantly with C-reactive protein levels, IL-6 and IFN-gamma levels in women with fertility disorders. In contrast, mDCs showed significant upregulation of CD1a during chlamydial infection and correlated significantly with IL-12 levels in Chlamydia positive fertile women. β-estradiol levels were significantly higher in women having fertility disorders as compared to fertile women and have significant correlations (r = 0.65; P < 0.05) with pDCs numbers, CD80 expression, IL-6 levels and IFN-gamma levels in these women.

**Conclusion:**

These results suggest that development of sequalae in some women can be a result of interplay of many factors including type of dendritic cell, co stimulatory molecule expression, cytokine secretion pattern and hormone levels.

## Background

*Chlamydia trachomatis *infections are the most prevalent sexually transmitted bacterial infections worldwide [1]. In India, a high prevalence rate of genital chlamydial infection has been reported among symptomatic women, of which upto 30% have fertility related disorders as multiple spontaneous abortions, infertility (not male related), or sub fertility [2,3]. Spontaneous clearance of *C. trachomatis *from the lower genital tract occurs in nearly 20% of chlamydial infections without any sequelae [4], however, in absence of treatment, these infections often recur, or remain persistent, leading to structural damage to the inflamed tissue and increase the risk of developing a sequelae [5,6]. Host factors such as type of immune response, plays a large role in determining the course and morbidity of *C. trachomatis *infections but the exact immunopathological mechanism leading to *Chlamydia *induced sequelae is still not well understood.

During chlamydial infection, T-cell mediated adaptive immune responses, play a major role in the clearance and resolution of infection [7]. T cells are activated by antigen presenting cells as dendritic cells (DCs) which play a crucial role in initiation and maintenance of T-cell immunity [8]. Besides providing protection, DCs have also been reported to be involved in chronic inflammation [9]. The cervical mucosa is reported to contain numerous DCs interdigitating between the epithelial cells [10] and these DCs often stain positive for CD1a [11]. DCs have also been reported to produce large amounts of interleukin-12 (IL-12) upon *ex vivo *pulsing with inactivated chlamydial organisms [12].

Two major DC subsets have been described; the CD123^+ ^DCs designated as plasmacytoid DCs (pDCs) and the CD11c^+^CD123^- ^(-/dim) designated as myeloid DCs (mDCs). the subsets express high levels of HLA-DR and lack the lineage markers CD3, CD14, CD19, CD20, CD16, and CD56, however, functional differences between the two have been described which include T-cell stimulatory activity, production of proinflammatory cytokines and differential expression of co-stimulatory molecules [13-15]. The expression of various co-stimulatory molecules (CD80/86) associated with DCs, have also been reported to modulate the type of immune response [16]. Further upon chlamydial infection, mononuclear cells are triggered to release a number of proinflammatory cytokines including TNF-α, IL-1, IL-6 and IL-8 [17,18] and many studies have reported association between cytokine profiles and immunopathogenic mechanisms. We have also reported previously that estradiol levels can modulate immune response in women with chlamydial infection [19] and can have a role in *Chlamydia *induced pathology.

In a recent study from our laboratory [20] we have reported that chlamydial infection recruits both mDCs and pDCs to the cervix and suggested that pDCs may be responsible for the immunopathogenesis of chlamydial infection, thereby, helping in the induction of sequelae. These results along with above facts prompted us to study the mobilization of these DC subsets in *Chlamydia *positive women, with or without fertility related disorders (multiple spontaneous abortions and infertility (not male related) and to evaluate the role played by these DC subsets in providing a protective/pathogenic immune response. We also measured the expression of various co-stimulatory molecules associated with these DCs and cytokines in cervical washes. C-reactive protein (CRP) levels and sex hormone levels in the sera of women with and without fertility disorders were also studied to understand the basic question in chlamydial pathogenesis as to why some women clear infection while others develop sequelae.

## Methods

### Study population

After obtaining informed written consent, 153 patients attending the gynecology outpatient department, Safdurjang Hospital, New Delhi, India were enrolled for the study. Twenty eight healthy age-matched controls attending the family planning department for birth control measures and with no previous history of any sexually transmitted disease (STD) were also enrolled. The study received approval from the hospital's ethics review committee. Procedures followed for sample collection were in accordance with the ethical standards for human experimentation established by the Declaration of Helsinki of 1975 (revised in 1983).

At recruitment, a detailed clinical questionnaire was administered to each patient for collecting information on reasons for referral, gynecology history including menstruation, symptoms of genital and urinary tract infection, obstetric and medical histories. Patients taking oral contraceptives, having positive urine pregnancy test, recent antibiotic therapy, and history of recently treated sexually transmitted infection and genital tuberculosis were excluded from the study.

Women with fertility related disorders included women with infertility and multiple spontaneous abortions. Infertile women where identified as those, which lack recognized conception after 1.5 to 2 years of regular intercourse without the use of contraception. Women with multiple spontaneous abortions (more than 2) have been described as those having delivery of pre-viable foetus before the 20^th ^week of gestation. Fertile women were those having last child birth within last 4 months to 1 year and testing positive for *C. trachomatis *during last pregnancy.

Since variations in sex hormones are known to influence cytokines concentrations and immune cell populations including the mDC and pDC [21], cervical samples were collected during mid-cycle (median 13 days, range 9th to 15th day of the menstrual cycle). None of the patient had sexual intercourse 3 days or more prior to collection of sample.

### Collection of samples

After cleaning the endocervix, endocervical swabs (HiMedia, Mumbai, India) were collected for diagnosis of *C. trachomatis *and other sexually transmitted disease (STD) pathogens. Cervical washes for determination of cervical cytokines were collected in 5 ml of sterile saline administered through a sterile Pasteur pipette and recovered after thorough washing of the cervix. For collection of cervical cells, a cytobrush was placed within the endocervical canal so that the cells from the endocervical region and the zone between the endocervical and ectocervical region (transformation zone) could be obtained. Cells were then transferred to a sterile tube containing sterile PBS (pH 7.2) supplemented with 100 U penicillin/mL, 100 μg streptomycin/mL, and 100 μg glutamine/mL. No samples were collected from patients with friable cervix and contact bleeding to ensure collection of cervical lymphocytes only. Heparinized peripheral venous blood (5 mL), was also collected after the diagnostic samples. Samples were then stored at 4°C until, they were transported to the laboratory and were then processed within 1 h.

### Microbiology

Samples were confirmed for chlamydial positivity by Direct Fluorescent Analysis (DFA) using fluorescein isothiocyanate (FITC) conjugated monoclonal antibodies to *C. trachomatis *major outer membrane protein (Microtrak, Palo Alto, USA). A sample was considered to be positive when greater than 10 elementary bodies (EB's) were detected. DFA negative samples were further confirmed for positivity by PCR analysis using primer specific for 517 bp plasmid of *C. trachomatis *[22]. Gram stained cervical smears were examined for the presence of yeast cells (Candidiasis) and quantification of polymorphonuclear leukocytes (PMNLs) per high-powered field (hpf) was done in smears to ensure presence of cervicitis. Vaginal smears were analyzed for clue cells, for diagnosis of bacterial Vaginosis. Wet mount microscopy was performed for the diagnosis of *Trichomonas vaginalis*. *Neisseria gonorrhoeae, Mycobacterium hominis *and *Ureaplasma urealyticum *were detected by culture as described previously [23].

### Isolation of cells from cervical samples

Cervical cells were isolated from the cytobrush by vigorously rotating it against the sides of the transport tube after incubating the sample with 5 mM DL-dithiothreitol (Sigma, St Louis, MO) at 37°C for 15 min (to reduce the mucus component of the sample). The cell suspension obtained was then filtered through a sterile 70 μm nylon cell strainer (BD Biosciences, San Diego, USA) and centrifuged at 300 *g *for 10 min; the resultant pellet yielding endocervical cells. Viability of cells was determined by trypan blue exclusion assay. The population of epithelial cells and lymphocytes in the cytobrush sample was counted with a haemocytometer and samples containing less than 1 million lymphocytes/ml were excluded.

### DC identification

**Figure 1:**
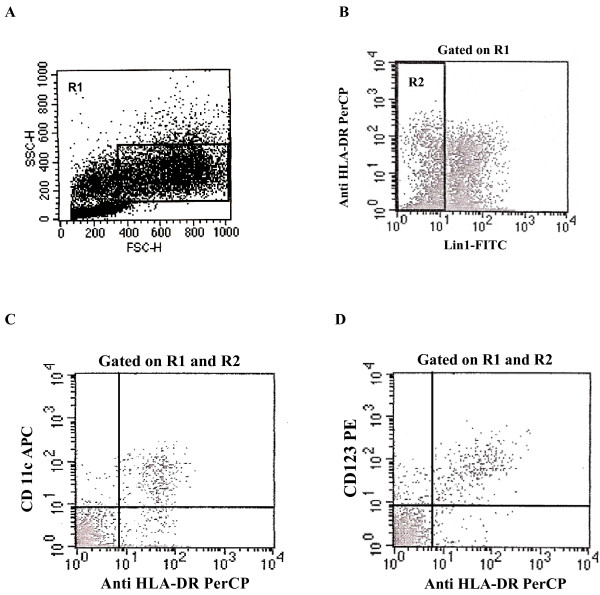
**Quantification of dendritic cell subsets in cervical mucosa by flow cytometric analysis.** (A) Introduction of an acquisition gate (R1) on the forward-scatter (FSC) versus side-scatter (SSC) profile to select mononuclear cell population. (B) cervical DCs were identified within gate R2 as lineage fluorescein isothiocyanate (FITC) cocktail (contains anti-CD3, CD14, CD16, CD19, CD20, and CD56) negative and HLA-DR positive. (C-D) Detection of HLA-DR^+ ^CD11c^+ ^myeloid DCs and HLA-DR^+^CD123^+ ^plasmacytoid DCs respectively. FSC, forward scatter; SSC, side scatter; PerCP, peridin chlorophyll protein; PE, phycoerythrin; APC, allophycocyanin.

For identification of DCs, following monoclonal antibodies (MoAb) were used: fluorescein isothiocyanate (FITC) conjugated lineage cocktail LIN-1 (anti-CD3, anti-CD14, anti-CD16, anti-CD19, anti-CD20 and anti-CD56), CD123-phycoerythrin, HLA-DR-peridin chlorophyll protein (BD Biosciences). In addition, FITC labeled anti-CD4, anti-CD8, anti-CD19 (for identification of CD4 T cells, CD8 T cells and B cells respectively) and allophycocyanin labeled anti-CD11c were purchased from eBiosciences (San Diego, USA) and PE labeled anti-CD80 and CD86 (costimulatory molecules involved in diferential activation of T-helper pathways) CD83 (DC maturation marker) and CD1a (immature DC marker) were obtained from BD Biosciences. To measure expression of CD80, 83, 86 and 1a on CD123^+ ^cells anti-CD123-PE-Cy5 and HLA-DR-APC were purchased from BD Biosciences. For blood dendritic cells, 100 μl of whole blood was incubated with antibody cocktail for 20 min at room temperature. Erythrocytes were lysed with FACS Lysing Buffer (BD Biosciences), cells were washed with PBS (with 0.1% (w/v) BSA and 0.1% NaN3) to remove unbound MoAb and resuspended in 1%(w/v) paraformaldehyde in PBS. For cervical cells, 100,000 cervical cells/tube were incubated with antibody cocktail for 25 min on ice and subsequently washed and fixed as per the protocol above. Cell preparations were labelled in parallel and included all appropriate isotype control antibodies (BD Biosciences) for establishing the demarcation between negative and positive populations. As previously described [19], cervical specimen exhibit high level of granularity and autofluorescence that could be attributed to many factors. Background fluorescence and presence of lymphocytes was minimized by introduction of an acquisition gate on the forward-scatter (FSC) versus side-scatter (SSC) profile, which included most of the monocytic and dendritic cell fraction and gave reliable differentiation of these cells from epithelial cells, lymphocytes and cell debris. Samples were acquired using a FACS Calibur Cytometer and analyzed with cell quest software (Becton Dickinson). DCs were identified as lineage FITC cocktail negative and HLA-DR positive population. The gating strategy used to identify and quantify LIN^-^/DR^+ ^cells is described in Figure 1.

mDCs and pDCs in cervical secretions were measured as number of events present per 100,000 cells taken for experiment. This number was then adjusted according to the total number of cells obtained in that cervical sample. These were finally presented as number of events per cervical sample. In case of blood the dendritic cell subsets were counted as events per 100 μl of blood taken and were then calculated and represented as events per milliliter of blood.

### Quantification of cytokines in cervical washes

Quantification of IL-4, IL-6, IL-8, IL-10, IL-12, TNF-α and IFN-γ was done using commercially available ELISA kits (eBiosciences, San Diego, CA), in accordance with the manufacturer's instructions. Briefly, 96-well ELISA plates were coated overnight with antihuman IL-4, IL-6, IL-10, IL-8, IL-12, TNF-α and IFN-γ capture antibodies. Unbound coating antibody was removed, and nonspecific protein binding sites were blocked with assay diluent as per the manufacturer's instructions. Duplicate serial dilutions of test samples or controls were incubated for 2 h at ambient temperature. Detector antibodies were added and incubated for 1 h, followed by incubation with peroxidase-conjugated antimouse IgG for 30 min. For color development, tetramethylene benzidine was added and then stopped after 15 min by the addition of 1 N sulfuric acid. Absorbance was read at 450 nm with reference absorbance of 650 nm. A log log standard curve was generated, and unknowns were interpolated. The sensitivities of cytokine kits were 1 pg/mL.

### Determination of C-reactive protein levels

C-reactive protein levels in sera were measured by commercially available hs-CRP ELISA kit (Calbiotech, USA) and levels above 2 microgram per millilter were considered to show higher risk of chronic inflammation as per the manufacturer's instructions.

### Hormonal assay

Levels of β-Estradiol and progesterone in sera of patients and controls were measured using commercially available ELISA kits (DRG, International Inc, USA) as per the manufacturer's instructions.

### Statistical analysis

The Kruskal-Wallis non parametric test was used to compare continuous variables among multiple groups. Categorical variables were compared using χ^2 ^test. Correlation was determined with spearman's correlation coefficient. P < 0.05 was considered to be significant.

## Results

### Study population

Cervical *C. trachomatis *infection was diagnosed by Direct Fluorescence Assay/PCR in 65 patients. Nine of these patients were co-infected either with *Candida *spp., bacterial vaginosis, *T. vaginalis*, *M. hominis*, *U. urealyticum*, or with *N. gonorrhoeae *and were thus excluded from the study. Two *Chlamydia *positive patients were excluded due to lower mononuclear cell count (less than 1 million cell/ml). *Chlamydia *positive women were divided into two groups: (a) *Chlamydia *positive fertile women (n = 34) and (b) *Chlamydia *positive women with fertility related disorders (n = 20). No significant differences in the median ages of the patients (fertile and with fertility disorders) or controls was observed (27, 27 and 28 years respectively).

### Immune cell population in the cervix

Flowcytometric analysis demonstrated the presence of mDCs, pDCs, CD14^+ ^monocytes, CD3^+^CD4^+ ^T cells and CD3^+^CD8^+ ^T cells in both the *Chlamydia *positive groups and controls. The median range of CD3^+^CD4^+ ^T cells and CD3^+^CD8^+ ^T lymphocytes among endocervical leucocytes in women which were included in the study was between 59% and 86% that of B lymphocytes was between 1% and 4%. Significant increase was seen in the mean number of CD4^+ ^T-lymphocytes per 10,000 events in cervical mucosa of fertile women compared to women with fertility disorders and controls (2195 *versus *401 and 262 respectively; P < 0.05). In contrast, the CD8^+ ^T cell population in cervical mucosa was found to be high during chlamydial infection but was non significant compared to controls. The number of CD14^+ ^monocytes per 10 000 events was found to be significantly increased in women with chlamydial infections compared to controls (324 *versus *312 and 147; P < 0.05 for fertile women, women with fertility disorders and controls respectively).

### mDC and pDC population in cervical mucosa and peripheral blood

The median and range of absolute numbers of mDCs and pDCs are given in Table 1. Healthy controls have significantly lower number of mDCs and pDCs in their cervical samples as compared to *Chlamydia *positive patient groups (P < 0.01). In comparison to cervical samples there was significant decrease in the absolute numbers of pDCs/mL of blood in women infected with *Chlamydia *when compared with controls. The decrease in pDC number in blood was found to be more pronounced in case of women with fertility disorders. In cervix of both the *Chlamydia *positive groups, the median number of pDCs was found to be higher than mDCs and was significant in case of women with fertility disorders (P < 0.05) (Table 1). In controls the median number of mDCs was higher to pDCs but the difference was not significant (Table 1).

**Table 1:** Absolute median numbers of LIN^-^DR^+ ^CD11c^+ ^(mDCs) and LIN^-^DR^+^CD123^+ ^(pDCs) dendritic cells in cervix (per cervical sample) and peripheral blood (per milliliter) of *Chlamydia trachomatis *positive and negative women.

		**CT Positive Fertile** (n = 34)	**CT Positive with Fertility Disorders** (n = 20)	**Control** (n = 28)
**mDCs**	**Blood**	6300 (276–24734)^¶^	8138 (175–17525)	15834 (3148–57180)
	**Cervix**	1254 (0–9634)^¶^	1856 (21–8473)^¶^	28 (0–437)
**pDCs**	**Blood**	6728 (4637–23820)^¶^	2645 (969–8657)	12857 (3051–45496)
	**Cervix**	1849 (0–50378)^¶^	3483 (54–10845)^¶#^	17 (0–195)

Note: CT-*Chlamydia trachomatis*; ^¶^P < 0.05 compared to controls; ^#^P < 0.05 compared to mDCs in cervix of *Chlamydia *positive women

### Expression of cell surface markers on mDC and pDC in cervical mucosa

In cervical mDCs of *Chlamydia *positive fertile women and on pDCs of *Chlamydia *positive women with fertility disorders, significantly high expression of CD80 was observed compared to the controls (P < 0.05). Expression of CD83 was significantly higher on both cervical mDCs and pDCs compared to controls. The expression of CD83 on pDCs obtained from women with fertility disorders was significantly higher than that of pDCs obtained from fertile women. CD86 expression on both mDCs and pDCs was found to be lower in *Chlamydia *positive women with or without fertility disorder, compared to controls but the difference was not significant (Table 2). Expression of CD1a on mDCs was significantly higher (P < 0.01) in *Chlamydia *positive groups as compared to controls (Table 2). The expression on pDCs of CD1a was higher in of *Chlamydia *positive groups compared to controls but was non significant.

**Table 2:** The percentage expression of various co-stimulatory molecules on the surface of mDCs and pDCs subsets in cervical mucosa.

	**% expression on mDCs**			**% expression on pDCs**		
	**CT+ Fertile** (n = 34)	**CT+ with FD** (n = 20)	**Controls** (n = 28)	**CT+ Fertile** (n = 34)	**CT+ with FD** (n = 20)	**Controls** (n = 28)
**CD80**	16.0 ± 3.0^a^	12.0 ± 2.2	7.0 ± 0.06	8.0 ± 0.7	32.5 ± 2.5^b^	6.0 ± 0.5
**CD83**	23.0 ± 0.6^a^	28.0 ± 1.8^a^	11.0 ± 2.0	18.0 ± 2.0^a^	38.0 ± 2.4^b^	8.0 ± 0.7
**CD86**	62.0 ± 7.0	59.0 ± 2.6	82.0 ± 5.0	21.0 ± 4.0	20.0 ± 1.9	31.0 ± 2.0
**CD1a**	43.0 ± 3.0^a^	37.0 ± 3.8^a^	18.0 ± 3.0	45.0 ± 3.0	41.0 ± 2.7	28.0 ± 1.0

Note: CT-*Chlamydia trachomatis*; FD-Fertility Disorders. The data are means ± SD. To study expression of various costimulatory molecules on mDC and pDC subsets, the gate was set on LIN^-^DR^+ ^cells, and LIN^-^DR^+ ^cells are again evaluated on quadrants having one axis as FL-2 (having Abs for costimulatory markers); ^a^P < 0.01 compared to controls; ^b^P < 0.01 compared to controls and CT positive fertile women.

### Concentration of cytokines in cervical washes and their correlation with DC populations

The median levels of IL-6, IL-8, IL-10, IL-12, TNF-α and IFN-γ in cervical washes of controls and *Chlamydia *positive women are shown in Table 3. Significantly higher levels of IL-6, IL-8, Il-10 and IFN-γ (P < 0.05) were observed in *Chlamydia *positive women with fertility disorders compared to *Chlamydia *positive fertile women and controls (Table 3). IL-12 levels were significantly higher (P < 0.05) in *Chlamydia *positive fertile women compared to both the other groups. No significant difference between TNF-α levels in different groups was observed (Table 3). IL-4 was below detectable limits in all the cases.

**Table 3:** Cytokine concentrations in cervical washes of Chlamydia positive women and controls.

	**Control**	**CT positive fertile women**	**CT positive women with fertility Disorder**
**IL-6**	34.97 (04.5–418.0)	19.83 (36.4–598.3)	74.47 (23.7–1674.8)^#^
**IL-8**	89.9 (UDL-247.57)	137.4 (13.7–375.9)	551.58 (32–1074.0)^#^
**IL-10**	07.57 (UDL-20.5)	09.27 (UDL-36.0)	27.94 (UDL-105.98)^#^
**IL-12**	127.16 (105.7–279.8)	294.62 (136.0–275.2)^#^	148.51 (89.5–602.9)
**IFN-γ**	115.91 (95.1–489.5)	164.37 (103.4-573.7)	347.96 (144.1–460.3)^#^
**TNF-α**	102.15 (34.8–356.1)	102.93 (59.8–418.4)	101.97 (93.2–507.0)

NOTE. Data represent median values. Figures in parentheses depict range. CT, *Chlamydia trachomatis*; Dis, Disorders; UDL-Under detection limit

^# ^P < 0.05 compared to other groups by Mann Whitney U test

Significant correlation (r = 0.65; P <0.05) was observed between number of mDCs/cervical sample and levels of IL-12 in *Chlamydia *positive fertile women. Number of pDCs/cervical sample in women with fertility disorders showed significantly high correlation with IL-6 levels (r = 0.78; P < 0.01) and IFN-γ levels (r = 0.58; P < 0.05). A less apparent correlation of IL-8 levels with pDCs/cervical sample in women with fertility disorders was observed (r = 0.41). CD80 expression on cervical mDCs from *Chlamydia *positive fertile women showed correlation with IL-12 levels (r = 0.56; P < 0.05). CD80 expression on pDCs from *Chlamydia *positive women with fertility disorders showed significant correlation with both IL-12 and IFN-γ levels (r = 0.73; P < 0.01 and r = 0.88; P = 0.001 respectively). CD86 levels showed significant correlation with IL-10 levels (r = 0.62; P < 0.05).

### Correlation of mDC and pDCs/cervical sample with C-reactive protein levels and sex hormone levels

Median CRP levels were found to be significantly higher in women with fertility disorders (Table 4) and showed significant correlation with pDCs/cervical sample (r = 0.63, P < 0.05). No correlation was found between number of mDCs/cervical sample and CRP levels. Estradiol or progesterone levels were considered positive if higher than > 2SD of the mean obtained from control patients. Number of estradiol positive women was significantly high among women with fertility disorders (65%, 13/20) compared to fertile women (15%, 5/34). Median estradiol levels were also found to be significantly higher in women with fertility disorders (Table 4). Estradiol levels further showed significant correlations with pDC numbers in women with fertility disorders (r = 0.65; P < 0.05), with CD80 expression (r = 0.74 and r = 0.58 for both *Chlamydia *positive women with or without fertility disorders respectively). IL-6 and IFN-γ levels also showed significant correlation with estradiol levels in women with fertility disorders (r = 0.49 and 0.73 respectively). No significant difference in the level of progesterone was found among different groups (Table 4) Further, no correlation was observed between progesterone levels and any of parameters studied.

**Table 4:** Levels of β-estradiol, CRP and Progesterone in serum of controls and Chlamydia positive women.

	**β-estradiol**	**CRP**	**Progesterone**
**Control**	205 (142–257)	1.06 (UD-2.05)	3.6 (0.2–4.8)
**Fertile**	153 (73–154)	1.13 (UD-2.27)	4.1 (0.1–6.1)
**Fertility Disorders**	207 (156–243)^#^	3.95 (0.57–11.84)^#^	3.8 (0.1–5.7)

**NOTE. **Data are median levels of β-estradiol and CRP in picograms per milliliter and nanogram per milliliter respectively. Figure in parentheses denote range.

^#^P < 0.05 compared to other groups

## Discussion

In women, chlamydial infections are often asymptomatic, and subsequent reinfections lead to inflammatory responses with pathological sequelae [24]. In peripheral circulation, majority of LIN^-^/DR^+ ^DCs cells express either CD11c or CD123 molecules [25,26], but no information is available on presence and role of these subsets during chlamydial infection. In this study, median number of pDCs in the cervix was found to be significantly higher than mDCs in both the *Chlamydia *positive groups. The number of cervical mDCs was significantly higher in fertile women in contrast to number of pDCs which were much higher in women with fertility disorders. These results depict that different pathological conditions are associated with different phenotype of dendritic cells and in women having sequelae to chlamydial infection, pDCs outnumber mDCs in the cervix. A previous study has also demonstrated enhanced number of pDC count in bronchoalveolar lavage fluid of immunocompetent patients with pneumonia [27]. Another study by Hartmann et al., have also shown high number of pDCs in patients with upper respiratory tract infection [28]. In comparison to mDCs, high number of pDCs were reported to be present in synovial fluid from patients with spondyloarthropathy [29]. We have also reported [20] higher number of mDCs in *Chlamydia *positive women with non-inflamed cervix compared to pDCs which were abundant in inflamed cervix. Beulens et al., have originally described mDCs as functionally mature DCs with a strong T cell stimulatory capacity [30] and have been reported to induce protective immune response [25], whereas, pDCs are generally defined as tolerogenic dendritic cells. Thus, these results collectively suggests that mDCs provide protective response while pDCs are involved in pathogenesis but how they help in development of a disorder is not well understood.

As for immune modulation by costimulatory molecules, previous studies have demonstrated that CD80/CD86 co-stimulatory molecules differentially activate T_H_1/T_H_2 type pathway and act as co-stimulatory signals for generation of T_H_1/T_H_2 cells CD4^+ ^T cells [30,31]. It has been shown previously that silencing of CD80 expression on dendritic cells significantly decreases IFN-γ secretion while that of CD86 decreases IL-4 secretion [32]. CD86 has also been shown to stimulate IL-10 production in CD4+ T cells [33]. Significantly high expression of CD80 molecules on pDCs obtained from women with fertility disorders and on mDCs from fertile women was observed. This suggests that significantly high expression of CD80 may lead to increased activation of T_H_1 cells during Chlamydial infection. As no difference in expression levels of CD86 among different groups was observed it suggests that CD86 may not be involved in induction of immune response to *C. trachomatis*.

Further, our results showed significantly higher expression of CD83 on both cervical mDCs and pDCs compared to controls. In comparison to mDCs the expression was significantly high on pDCs from women with fertility disorders. CD83 expression is taken as a marker for phenotypic maturation of dendritic cells and higher percentage of DCs expressing them in both *Chlamydia *positive cases show that during chlamydial infection high number of both mDC and pDC take up antigen for processing and turn into mature DCs. This data suggests that although both mDCs and pDCs are present in the cervix of women with fertility disorders but still pDCs are the ones which are more matured and hence activated.

When cervical washes were studied for cytokine concentrations it was seen that in *Chlamydia *positive women with fertility disorders significantly higher levels of IL-6, IL-8, IL-10 and IFN-γ were present compared to the other groups. In comparison to this IL-12 levels were significantly higher in *Chlamydia *positive fertile women. Although not as protective as IFN-γ, IL-6 (generated either by epithelial cells or by the interaction of chlamydiae with T lymphocytes) is probably important, together with IL-12, for sustaining the protective T-helper 1 cell mediated immune response [34]. Although protective in nature, pathogenic role of IL-6, has been shown previously [35,36]. In earlier study increased IL-6 levels during in silent tubal infections of *C. trachomatis *have been reported [37]. As for IL-8, it has also been shown previously that synovial tissues from chronic arthritis patients with synovial *C. pneumoniae *infection have significant levels of mRNA for IL-8 [38]. These results suggest that IL-6 and IL-8 may be involved in chlamydial pathology but their actual role and mechanism is yet to be ascertained.

IL-10 was found to be up-regulated in cervical washes obtained from *Chlamydia *positive women with fertility disorders. IL-10 is not always an inflammatory/inhibitory cytokine; instead higher levels of IL-10 probably prevent the pathological effect of the inflammatory cytokines like IL-1β, IFN-γ and TNF-α. IL-10 can create a favourable environment for persistence of microbes by down-regulating the proinflammatory cytokines. In case of chlamydial infection IL-10 has been reported to be associated with typical pathological changes like fibrosis and granuloma formation [39]. In case of ocular chlamydial infection, IL-10 has been shown to be associated with scarring and blindness [40]. Association of increased frequency of IL-10 detection in endocervical secretions of women with non-ulcerative STDs, like *C. trachomatis *have suggested that this may be a potential mechanism through which these infections may alter susceptibility to HIV-1 infection [41]. These results along with ours, suggests, that excessive secretion of IL-10 may lead to either incomplete clearance of bacteria or may develop a state of anergy resulting in development of fertility related disorders.

IL-12, on the other hand was significantly higher in *Chlamydia *positive fertile women compared to other groups. IL-12 is derived from dendritic cells and monocytes and it induces T_H_1 differentiation with induction of IFN-γ [42]. These results suggest that high secretion of IL-12 may be responsible for providing a protective immune response to chlamydial infection.

IFN-γ levels were found to be significantly higher in women with fertility disorders as compared to other groups. A previous study by our group [19] have shown also high levels of IFN-γ in cervical washes of women with recurrent chlamydial infections compared to women with primary chlamydial infections and has demonstrated that high secretion of pro-inflammatory cytokines as IL-6, IFN-γ and TNF-α lead to acute inflammation and can thus cause reproductive disorders. An earlier study by Van Voorhis *et al*., 1997 has also shown that repeated chlamydial infection in a *Macca nemestrina *model high levels of IFN-γ transcripts were produced [43]. Further, in synovial tissue of patients with *C. trachomatis *associated arthritis, both IL-10 and IFN-γ producing cells were detected and it was suggested that excessive IL-10 production suppresses IFN-γ and mediated persistence [44].

High expression of CD80 on mDCs and its significant correlation with IL-12 levels in fertile women further suggest that the major role of mDCs is in clearance of the organism from the genital tract. On the other hand, CD80 expression on pDCs of women with fertility disorders showed significant correlation with both IL-12 and IFN-γ levels suggesting the role of CD80 activation in secretion of type 1 cytokines. Further, these results suggest that modulation of cytokine expression can be the main mechanism which decides whether the infection is cleared or will go for pathological damage. This would however, be hypothetical as there is no confirmation that in women with fertility related disorders the clinical condition is due to chlamydial infection and not due to any other cause. The results we got above may shed a new light on chlamydial pathogenesis but these have their limitations as; (1) scarcity of cell numbers making it impossible for us to separate these subsets and then see the expression of cytokines upon stimulation with *C. trachomatis *as increased number of pDCs can act as a factor acting to limit bacterial load and pathogenesis (2) the fact that cytokines like IL-6, IL-8 and IL-12 are secreted by other cell populations like macrophages and epithelial cells which outnumber DCs in the cervix.

Further, median CRP levels were found to be significantly higher in women with fertility disorders and showed significant correlation with pDCs. CRP levels are considered marker for inflammation and its association with pDCs in women with fertility disorders suggests that pDCs are involved either in induction or in maintenance of inflammatory responses.

We detected significantly high levels of estradiol in women with fertility disorders, which correlated significantly with pDC levels, CD80 expression and IL-6 and IFN-γ secretion. In a previous study in humans it has been shown that women are more susceptible to chlamydial infection under β-estradiol influence, since more chlamydial organisms can be isolated during the proliferative part of the cycle [45]. Estrogen was also found to enhance chlamydial adherence or intracellular development of their inclusion [46]. This suggests that estradiol may enhance inflammation by helping in increased secretion of proinflammatory cytokines either by up-regulating CD80 expression or by modulating the cytokine secretion profile of other cell. A previous study by Dimayuga et al., [47] has also shown up-regulation of CD80 expression in LPS stimulated microglial cells by estrogen treatment. Overall these results show that high estradiol levels may help in persistence of *Chlamydia *directly by helping in its intracellular development or indirectly by modulation of cytokine secretion profile of immune cells.

## Conclusion

Overall, our data suggests that a differential activation of subsets of DC, cytokine secretion pattern and hormonal levels can be responsible for modulating the immune response to chlamydial infection, but, the question that still remains unanswered is the mechanism by which these are modulated during chlamydial infections and this deserves further elucidation. Since there is an urgent need to develop a vaccine against chlamydial infection, therefore, the basic research challenge is to understand the requirements for inducing and maintaining protective genital mucosal immunity.

## Competing interests

The authors declare that they have no competing interests.

## Authors' contributions

TA participated in the design of the study, carried out experimental analysis, performed the statistical analysis and drafted the manuscript. VV participated in the design of the study, carried out flowcytometry and immunoassays. PKW conceptualised the idea, helped in drafting the manuscript and revising it critically for important intellectual conten. SS participated in the design of the study, helped in sample collection and helped in drafting of manuscript. AM conceived of the study, participated in its design and coordination and helped to draft the manuscript. All authors read and approved the final manuscript.

## References

[B1] Gerbase AC, Rowley JT, Mertens TE (1998). Global epidemiology of sexually transmitted diseases. Lancet.

[B2] George JA, Panchatcharam TS, Paramasivam R, Balasubramaniam S, Chakrapani V, Murugan G (2003). Evaluation of diagnostic efficacy of PCR methods for *Chlamydia trachomatis *infection in genital and urine specimens of symptomatic men and women in India. Jpn J Infect Dis.

[B3] Morrison RP, Caldwell HD (2002). Immunity to murine genital chlamydial infection. Infect Immun.

[B4] Parks KS, Dixon PB, Richy CM, Hook EW (1997). 3rd. Spontaneous clearance of *Chlamydia trachomatis *infection in untreated patients. Sex Trans Dis.

[B5] Golden M, Schillinger J, Markowitz L, St Louis M (2000). Duration of untreated genital infections with *Chlamydia trachomatis*: a review of the literature. Sex Transm Dis.

[B6] Schachter J (1978). Chlamydial Infections. N Engl J Med.

[B7] Johnson RM (2004). Murine oviduct epithelial cell cytokine responses to *Chlamydia muridarum *infection include interleukin-12-p70 secretion. Infect Immun.

[B8] Steinmann RM (1991). The dendritic cell system and its role in immunogenecity. Annu Rev Immunol.

[B9] Sallusto F, Lanzavecchia A (1999). Mobilizing dendritic cells for tolerance, priming and chronic inflammation. J Exp Med.

[B10] Pudney J, Quayle AJ, Anderson DJ (2005). Immunological microenvironments in the human vagina and cervix: mediators of cellular immunity are concentrated in the cervical transformation zone. Biol Reprod.

[B11] Prakash M, Kapembwa MS, Gotch F, Patterson S (2004). Chemokine receptor expression on mucosal dendritic cells from endocervix of healthy women. J Infect Dis.

[B12] Lu H, Zhong G (1999). Interleukin-12 production is required for chlamydial antigen pulsed dendritic cells to induce protection against live *Chlamydia trachomatis *infection. Infect Immun.

[B13] Reid SD, Penna G, Adorini L (2000). The control of T-cell responses by dendritic cell subsets. Curr Opin Immunol.

[B14] Thomas R, Lipsky PE (1994). Human peripheral blood dendritic cell subsets. Isolation and characterization of precursor and mature antigen-presenting cells. J Immunol.

[B15] Willmann K, Dunne JF (2000). A flow cytometric immune function assay for human peripheral blood dendritic cells. J Leukoc Biol.

[B16] Levine BL, Ueda Y, Craighead N, Huang ML, June CH (1995). CD28 ligands CD80 (B7-1) and CD86 (B7-2) induce long-term autocrine growth of CD4+ T cells and induce similar patterns of cytokine secretion in vitro. Inter Immunol.

[B17] Heinemann RE, Susa M, Simnacher U, Marre R, Essig A (1996). Growth of *Chlamydia pneumoniae *induces cytokine production and expression of CD14 in a human monocytic cell line. Infect Immun.

[B18] Redecke V, Dalhoff K, Bohnet S, Braun J, Maass M (1998). Interaction of *Chlamydia pneumoniae *and human alveolar macrophages: infection and inflammatory response. Am J Respir Cell Mol Biol.

[B19] Agrawal T, Vats V, Wallace PK, Salhan S, Mittal A (2007). Cervical cytokine responses in women with primary or recurrent chlamydial infection. J Interferon Cytokine Res.

[B20] Agrawal T, Vats V, Wallace PK, Singh A, Salhan S, Mittal A Recruitment of myeloid and plasmacytoid dendritic cells in cervical mucosa during *Chlamydia trachomatis *infection. Clin Microbiol Infect.

[B21] Zarnani AH, Moazzeni SM, Shokri F, Salehnia M, Jeddi Tehrani M (2006). Analysis of endometrial myeloid and lymphoid dendritic cells during mouse estrous cycle. J Reprod Immunol.

[B22] Singh V, Rastogi S, Garg S, Kapur S, Kumar A, Salhan S, Mittal A (2002). Polymerase chain reaction for detection of endocervical *Chlamydia trachomatis *infection in Indian women attending Gynecology out patient department. Acta Cytol.

[B23] Vats V, Agrawal T, Salhan S, Mittal A (2007). Primary and secondary immune response of mucosal and peripheral lymphocytes during *Chlamydia trachomatis *infection. FEMS Immunol Med Microbio.

[B24] Bailey RL, Holand MJ, Whittle HC, Mabey DC (1995). Subject recovering from human ocular chlamydial infection have enhance lymphoproliferative responses to chlamydial antigens compare with those of persistently diseased controls. Infect Immun.

[B25] O'Doherty U, Peng M, Gezelter S, Swiggard WJ, Betjes M, Bhardwaj N, Steinman RM (1994). Human blood contains two subset of dendritic cells, one immunologically mature and the other immature. Immunology.

[B26] Savary CA, Grazziutti ML, Melichar B, Przepiorka D, Freedman RS, Cowart RE, Cohen DM, Anaissie EJ, Woodside DG, McIntyre BW, Pierson DL, Pellis NR, Rex JH (1998). Multidimensional flow-cytometric analysis of dendritic cells in peripheral blood of normal donors and cancer patients. Cancer Immunol Immunother.

[B27] Lommatzsch M, Bratke K, Bier A, Julius P, Kuepper M, Luttmann W, Virchow JC (2007). Airway dendritic cell phenotypes in inflammatory diseases of the human lung. Eur Respir J.

[B28] Hartmann E, Graefe H, Hopert A, Pries R, Rothenfusser S, Poeck H, Mack B, Endres S, Hartmann G, Wollenberg B (2006). Analysis of plasmacytoid and myeloid dendritic cells in nasal epithelium. Clin Vaccine Immunol.

[B29] Van Krinks CH, Matyszak MK, Gaston JSH (2004). Characterization of plasmacytoid dendritic cells in in flammatory arthritis synovial fluid. Rheumatology.

[B30] Buelens C, Willems F, Delvaux A, Pierard G, Delville J, Velu T, Goldman M (1995). Interleukin-10 differentially regulates B7-1 (CD80) and B7-2 (CD86) expression on human peripheral blood dendritic cells. Eur J Immunol.

[B31] Kuchroo VK, Das MP, Brown JA, Ranger AM, Zamvil SS, Sobel RA, Weiner HL, Nabavi N, Glimcher LH (1995). B7-1 and B7-2 costimulatory molecules activate differentially the Th1/Th2 developmental pathways: Application to autoimmune disease therapy. Cell.

[B32] Suzuki M, Zhang X, Zheng X, Vladau C, Li M, Chen D, Gracia B, Min W (2007). Immune modulation through silencing CD80 and CD86 in dendritic cells using siRNA. J Immunol.

[B33] Nakajima A, Watanabe N, Yoshino S, Yagita H, Okumura K, Azuma M (1997). Requirement of CD28-CD86 co-stimulation in the interaction between antigen-primed T helper type 2 and B cells. Inter Immunol.

[B34] Yu JL, Yu P, Li LX (2003). HeLa cells secrete interleukin-8 and interlukin-10 response to *Chlamydia trachomatis *entry. Hunan Yi Ke Da Xue Xue Bao.

[B35] Mpiga P, Mansour S, Morisset R, Beaulieu R, Ravaoarinoro M (2006). Sustained interleukin-6 and interleukin-8 expression following infection with *Chlamydia trachoamtis *serovar L2 in a HeLa/THP-1 cell co-culture model. Scand J Immunol.

[B36] Darville T, O'Neill JM, Andrews CW, Nagarajan UM, Stahl L, Ojcius DM (2003). Toll-like receptor-2, but not toll-like receptor-4, is essential for development of oviduct pathology in chlamydial genital tract infection. J Immunol.

[B37] Li H, Liang Z (2000). Determination of tumour-necrosis factor-alpha and interleukin-6 levels of the tubal fluids in patients with infertility caused by infection of *Chlamydia trachomatis*. Zhonghua Fu Chan Ke Za Zhi.

[B38] Gerard HC, Wang Z, Whittum-Hudson JA, El-Gabalawy H, Goldbach-Mansky R, Bardin T, Schumacher HR, Hudson AP (2002). Cytokine and chemokine mRNA produced in synovial tissue chronically infected with *Chlamydia trachomatis *and *C. pneumoniae*. J Rheumatol.

[B39] Conti P, Kempuraj D, Kandere K, Di Gioacchino M, Barbacane RC, Castellani ML, Felaco M, Boucher W, Letourneau R, Theoharides TC (2003). IL-10, an inflammatory/inhibitory cytokine but not always. Immunol Lett.

[B40] Natividad A, Holland MJ, Rockett KA, Forton J, Faal N, Joof HM, Mabey DC, Bailey RL, Kwiatkowski DP (2008). Susceptibility to sequalae of human ocular chlamydial infection is associated with allelic variation in IL10 cis-regulation. Hum Mol Genet.

[B41] Cohen CR, Plummer FA, Mugo N, Maclean I, Shen C, Bukusi EA, Irungu E, Sinei S, Bwayo J, Brunham RC (1999). Increased interleukin-10 in the endocervical secretions of women with non-ulcerative sexually transmitted diseases: a mechanism for enhanced HIV-1 transmission?. AIDS.

[B42] Holland MJ, Bailey RL, Conway DJ, Culley F, Miranpuri G, Byrne GI, Whittle HC, Mabey DC (1996). T helper type-1 (Th1)/Th2 profiles of peripheral blood mononuclear cells (PBMC); responses to antigens of *Chlamydia trachomatis *in subjects with severe trachomatous scarring. Clin Exp Immunol.

[B43] Van Voorhis WC, Barrett LK, Sweeney YT, Kuo CC, Patton DL (1997). Repeated *Chlamydia trachomatis *infection of *Macaca nemestrina *fallopian tubes produce a Th1-like cytokine response associated with fibrosis and scarring. Infect Immun.

[B44] Kotake S, Schumacher HR, Arayssi TK, Gérard HC, Branigan PJ, Hudson AP, Yarboro CH, Klippel JH, Wilder RL (1999). Gamma-interferon and interleukin-10 gene expression in synovial tissues from patients with early stages of *Chlamydia*-associated arthritis and undifferentiated oligoarthritis and from healthy volunteers. Infect Immun.

[B45] Dimayuga FO, Reed JL, Carnero GA, wang C, Dimayuga ER, Dimayuga VM, Perger A, Wilson ME, Keller JN, Bruce-Keller AJ (2005). Estrogen and brain inflammation: effects on microglial expression of MHC, costimulatory molecules and cytokines. J Neuroimmunol.

[B46] Sweet RL, Blankfort-Doyle M, Robbie MO, Schachter J (1986). The occurrence of chlamydial and gonococcal salpingitis during the menstrual cycle. JAMA.

[B47] Bose SK, Goswami PC (1986). Enhancement of adherence and growth of *Chlamydia trachomatis *by estrogen treatment of HeLa cells. Infect Immun.

